# Brachytherapy emulating robotic radiosurgery in patients with cervical carcinoma

**DOI:** 10.1186/1748-717X-8-109

**Published:** 2013-05-02

**Authors:** Simone Marnitz, Christhardt Köhler, Volker Budach, Oliver Neumann, Anne Kluge, Waldemar Wlodarczyk, Ulrich Jahn, Bernhard Gebauer, Markus Kufeld

**Affiliations:** 1 Department of Radiooncology, Charité University Medicine Berlin, Augustenburger Platz 1, 13353 Berlin, Germany; 2 Department of Gynecology, Charité University Medicine Berlin, Charitéplatz 1, Berlin, 10117, Germany; 3 Institute for Radiology, Charité University Medicine Berlin, Augustenburger Platz 1, Berlin, 13353, Germany; 4 CyberKnife Center Charité, Charité University Medicine Berlin, Augustenburger Platz 1, Berlin, 13353, Germany

**Keywords:** Robotic radiosurgery, Cervical cancer, Boost, Emulating brachytherapy, CyberKnife

## Abstract

**Purpose:**

To evaluate the technique, dosimetry, dose-volume-histograms (DVHs) and acute toxicity for CyberKnife^®^ boost irradiation instead of intra-cervical brachytherapy in patients with cervical cancer.

**Methods and materials:**

Eleven who were not suitable for brachytherapy with FIGO stage IIB-IIIB cervical cancer underwent primary chemoradiation. After fiducial implantation, T2 contrast-enhanced planning MRI and CT scans at 2-mm slice thickness were collected in the treatment position. The clinical target volume was defined as cervix + macroscopic residual tumour on MRI. Five fractions of 6 Gy each were prescribed to the target volume with a covering single dose 6 Gy. DVH parameters were evaluated for the target and organs at risk. Acute toxicity was documented once a week.

**Results:**

Dmean_PTV_ ranged from 33.6-40 Gy, median 36.7 Gy with a coverage of the PTV calculated to 100% of the prescribed dose ranging from 93.0-99.3% (median 97.7%). For the PTV the median CN was 0.78 (range, 0.66 to 0.87) and the median CI was 1.28 (range 1.15 to 1.52). Gastrointestinal (GI) and genitourinary (GU) toxicity was mild. There was no grade 3 or higher GI and GU toxicity. After 6 months of follow up, there were no local recurrences. For the complete treatment, a median EQD2 to 1 cc and 2 cc of the bladder wall was 98.8 Gy and 87.1 Gy, respectively. Median EQD2 to 1 cc and 2 cc of the rectal wall was 72.3 Gy and 64 Gy, respectively, correlating with a risk < 10% for Grade 2–4 late toxicity.

**Conclusions:**

CyberKnife robotic radiosurgery in patients with cervical cancer provides excellent target coverage with steep dose gradients toward normal tissues and safe DVH parameters for bladder, rectum and sigmoid. Acute toxicity was mild. Longer follow-up is needed to evaluate the oncological equality.

## Introduction

Chemoradiation is the treatment of choice for patients with locally advanced and/or lymph-node positive cervical carcinoma. Local and loco-regional control remains a challenge in the treatment of cervical cancer patients. More than half of the patients who recur after chemoradiation have had a component of in-field failure.

Intra-cervical brachytherapy has been accepted as an integral part of curative primary chemoradiation. There is a wide range of techniques, dose concepts and schedules for brachytherapy, which complicates a comparison of oncologic results, treatment toxicity and applied doses. The concept of intra-cervical brachytherapy has changed during the last decade. Since the publication of the GEC-ESTRO Group recommendations, target volume definition, prescribing and documenting of dosage, fractionation and dose limits to organs at risk for brachytherapy have become more and more standardized [1]. Radiation doses delivered to 1 cc and 2 cc of the rectal wall have been found to be predictive for late rectal toxicity, whereas for the sigmoid and bladder do not exist accepted dose-volume relationships for toxicity [2]. However, a survey among gynaecological cancer intergroup clinics (GCIG) [3] showed that MRI guided brachytherapy was used by only 25% of the clinics.

Boost dose delivered using conventional external beam radiotherapy (EBRT) devices have, compared to brachytherapy, compromised disease control compared with brachytherapy [4]. Perhaps due to these disappointing findings, only a few groups have evaluated the feasibility and potential of highly conformal external-beam techniques for boosting small sub-volumes for patients with gynaecological carcinoma [5]–[10]. The introduction of several modern radiation technologies, such as image-guided radiotherapy (IGRT), stereotactic body irradiation, intensity modulated radiotherapy (IMRT), and volumetric arc therapy (VMAT) may allow the emulation of brachytherapy-like dose distributions, delivering high doses to the target without exceeding constraints to the organs at risk. Image-guided radiosurgery may be most suited to the present application—frameless, robotic targeting and tracking algorithms have allowed what was once soley an intracranial treatment to be extended to extracranial indications.

The CyberKnife^®^ radiosurgery system (Accuray Inc., Sunnyvale, CA) combines a 6-MV compact linear accelerator (1000 MU/min LINAC), mounted on a computer-controlled six-axis robotic manipulator, with a robotic treatment couch, which can move in three translational as well as three rotational directions. Its application throughout the body is made possible by its image-guided tracking system: a pair of orthogonally positioned x-ray sources and detectors acquires images during treatment at given time intervals that are registered to synthetic images derived from the planning CT volume. Offsets from the patient’s planning pose are used to automatically reposition the LINAC. The technology has been used successfully to treat lung cancer, liver metastases, CNS tumours and metastases as well as recurrent and oligo-metastatic gynaecologic tumours [8].

Robotic radiosurgery allows continuous tracking of the cervix during treatment using implanted markers, resulting in superior accuracy in dose delivery relative to EBRT. No general anaesthesia or smit sleeve insertion are required as for brachytherapy, and there is no intra-vaginal manipulation, no pain, and no need for analgesics. Patients do not need to be transported to the planning MRI with applicators. Finally, variability among physicians in technique (e.g., vaginal packing) and skill levels are minimized. The use of robotic radiosurgery was reported once previously; various EBRT and boost doses were tested on six patients with acceptable toxicity [11]. The aim of the present study was to evaluate the technique, dosimetry and acute toxicity following use of the CyberKnife to deliver a brachytherapy-like boost to patients with cervical cancer. To the best of our knowledge, the present study is the first test of a uniform dose concept for both brachytherapy emulation and EBRT.

## Methods and materials

**Figure 1:**
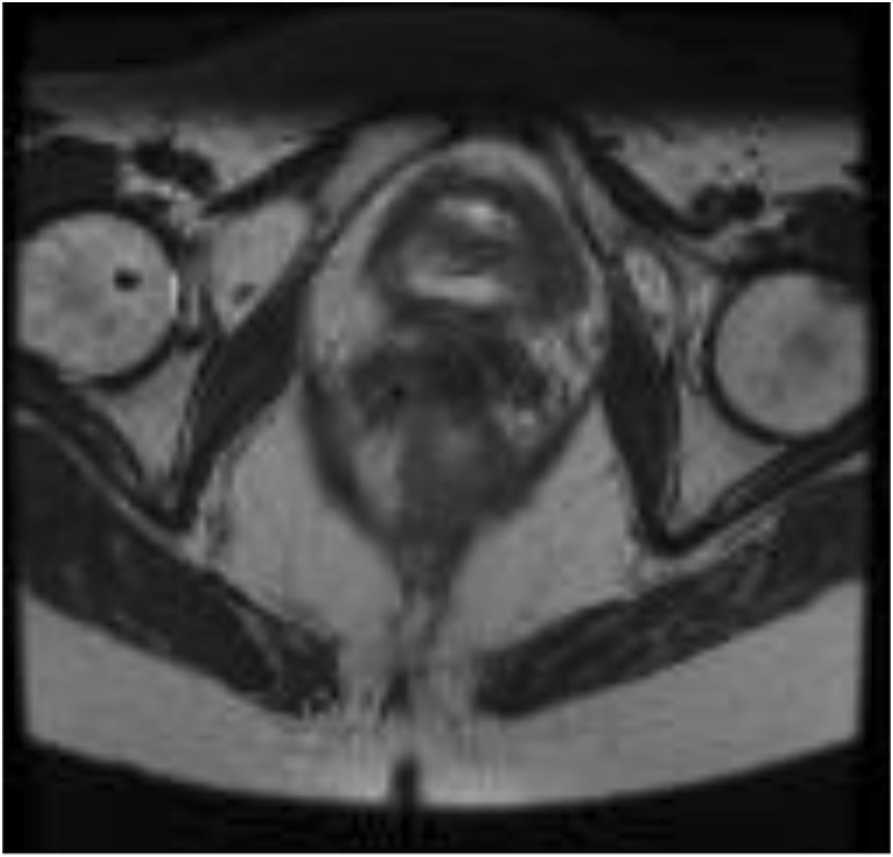
Transverse T2 MRI of the patient with uterus bicornis and bicollis and two cervical ossa.

From 2011–2012, eleven patients (32–69 years, mean 53.5 years) with histologically proven cervical cancer (squamous cell carcinoma, n = 8; adenocarcinoma, n = 3) underwent primary chemo-radiation. Nine patients presented with FIGO IIB, two with FIGO IIIB tumours. Indications for brachytherapy-emulating CyberKnife treatment were uterus bi-collis and bi-cornis (n = 1; Figure 1), refusal of smit sleeve insertion (n = 3) or refusal of brachytherapy (n = 7) (Figure 1). Patients receive detailed information and explanations in consultation with the investigating physician before being included in the study. The study has been approved by the IRB.

Prior to chemoradiation, three patients underwent transperitoneal surgical staging including pelvic and/or para-aortic lymphadenectomy. Two patients had no lymph node metastases, one patient showed histologically proven lymph node metastases with extra-capsular spread (9/20). Eight patients were staged clinically. A computed tomography (CT) of the abdomen did not show enlarged pelvic or para-aortic lymph nodes in any patient. The therapy consisted of EBRT with a simultaneous integrated boost as described elsewhere [12]. External beam radiation was performed with a linear accelerator with 6MV-photons. Five weekly fraction of 1.8 Gy single dose were prescribed to PTV-A (pelvic +/-para-aortic lymph nodes, uterus, cervix, parametric region) to a total dose of 50.4 Gy. A simultaneous boost was given to the parametric region with five weekly single doses of 2.12 Gy to a total dose of 59.36 Gy. Simultaneous chemotherapy was administered once weekly with cisplatin, 40 mg per square meter body surface (n = 9), carboplatin AUC 1.5 once weekly, five applications (n = 1) and one application cisplatin followed by four weekly applications carboplatin AUC 1.5 (n = 1) because of renal dysfunction after the first cisplatin application.

### Fiducial insertion

**Figure 2:**
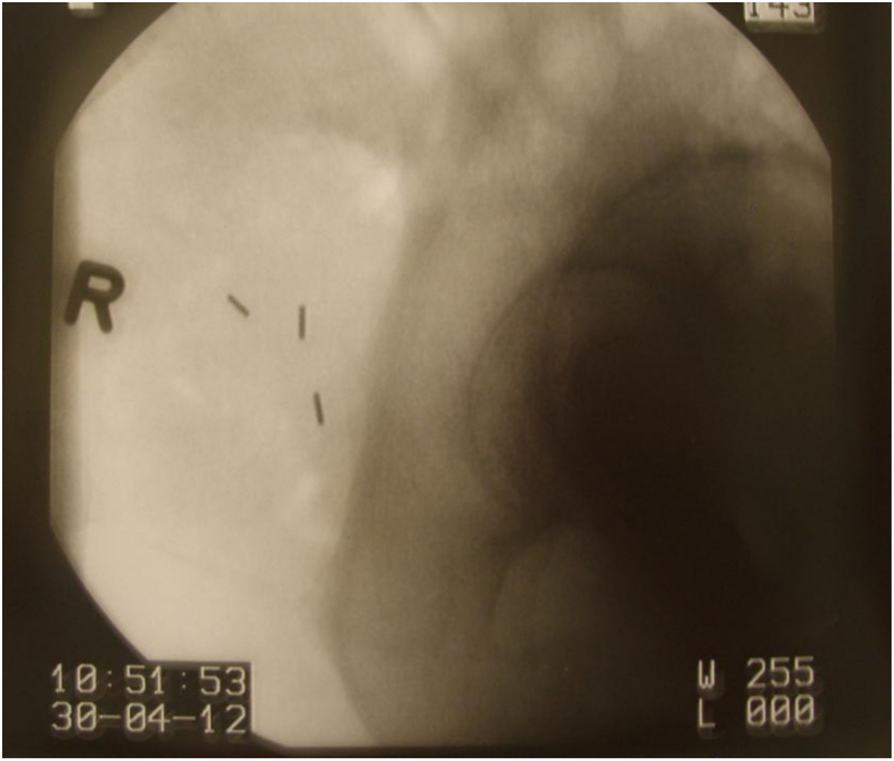
Fiducal insertion with Mic-Goldmarkers (5 mm/1 mm).

After three weeks of conventional EBRT, three to four Mick^®^-Fiducials (mps-Medical Physics Solutions, Melle, Germany), 1 mm in diameter and 5 mm in length, were placed in and around the tumor transvaginally (Figure 2). under visual guidance and fluoroscopic control using pre-loaded needles At least three fiducials were placed laterally into the cervix and in the medial anterior and/or posterior part.

### Contouring and target volume

MRI and CT image sets were fused based on bony landmarks and fiducials. The clinical target volume was defined as the cervix plus the macroscopic residual tumour in the cervix as identified on T2 MRI, including the whole cervix and the visible tumor on MRI outside the cervix. As is true for brachytherapy, no PTV margin was added.

#### Dose constraints for the organs at risk (OARs)

Rectal wall, bladder wall and sigmoidal wall were contoured as the organ’s outer wall minus 2 mm for the inner wall. The small bowel was defined as the whole peritoneal cavity excluding other organs at risk, muscles and the planning target volume (PTV) up to the 4th lumbar vertebra. Biologically effective doses from EBRT and CyberKnife were calculated with α/β = 3 for normal tissue. For CyberKnife Boost, organ walls of rectum, sigmoid and bladder were generated. The BED2Gy (α/β = 3) to 0.1, 2 and 5 cc of the rectal wall, bladder wall and sigmoid wall were calculated from DVHs from EBRT + CyberKnife according to Georg et al. [2]. For summarizing the EQD2 from CyberKnife and EBRT, the rectal wall, sigmoid wall and bladder wall was contoured in the planning CT and the dose were calculated as described above and added to those from CyberKnife. For EBRT (IMRT, Helical Tomotherapy or Rapid Arc), the following dose constraints were used for the whole organ: Small bowel: V45 < 20%, V20 < 40%, D_mean_ < 30 Gy; rectum: V40 < 70%, V50 < 50%; bladder: V30 < 60%, V50 < 30%; femoral heads: D_mean_ < 40Gy.

#### Dose prescription

**Figure 3:**
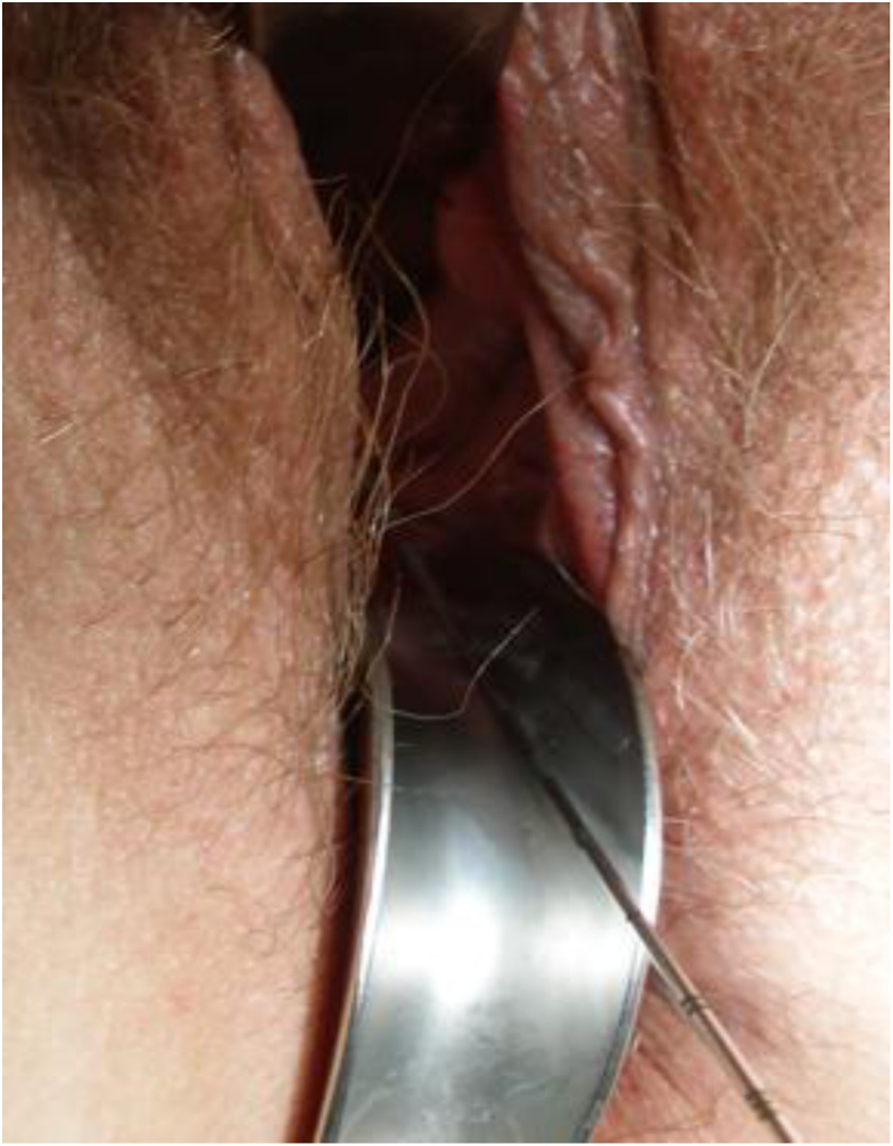
**Beam geometry for CyberKnife boost. **Sagittal view of the dose distribution (lower left image; 6 Gy isodose in pink, rectum in green, bladder in blue). Dose volume histogram in upper right panel.

**Figure 4:**
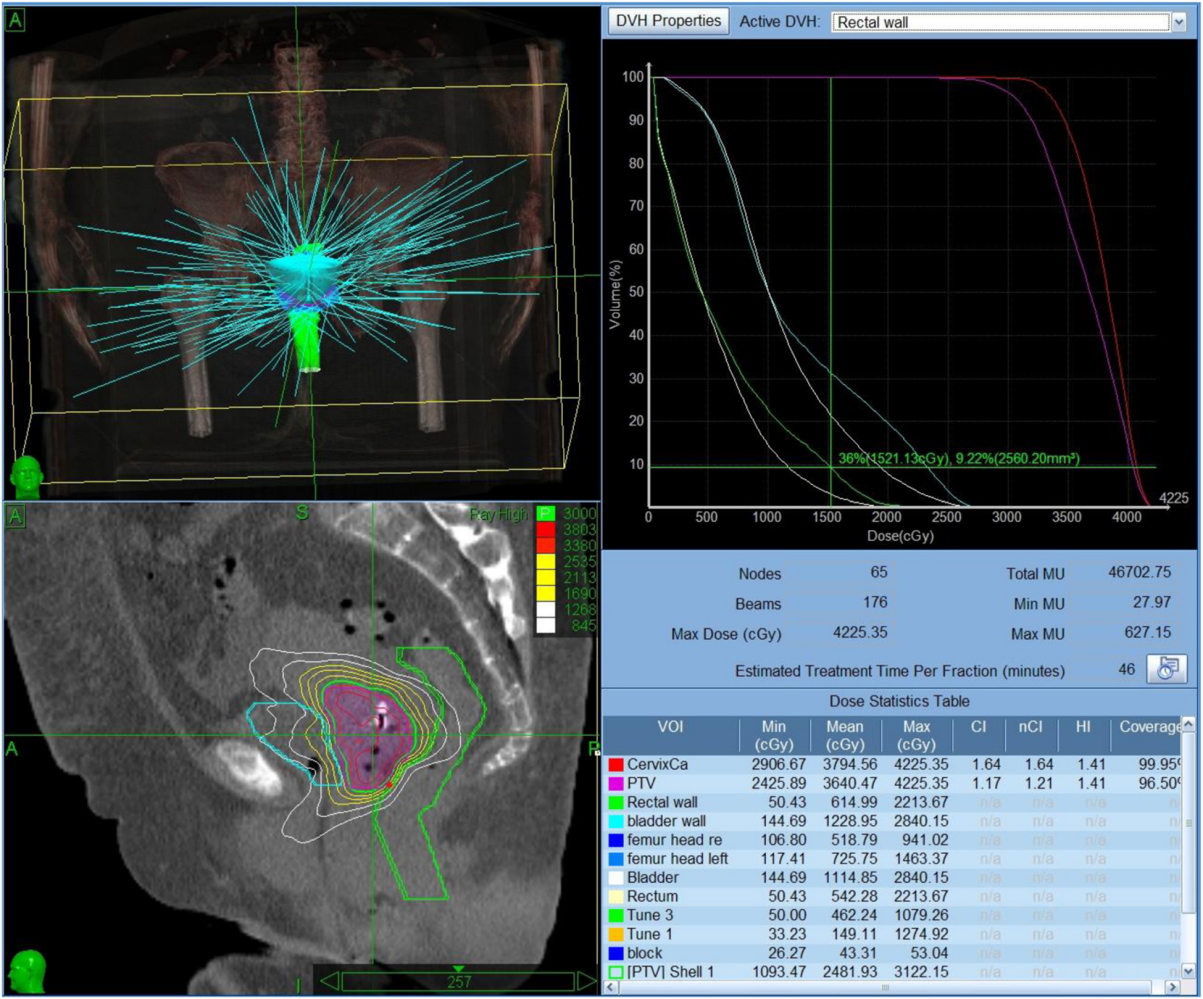
**Beam geometry for CyberKnife boost. **Sagittal view of the dose distribution (lower left image; 6 Gy isodose in pink, rectum in green, bladder in blue). Dose volume histogram in upper right panel.

Five single doses of 6 Gy were prescribed to the target volume. All fractions were given with an inter-fraction interval of at least 72 h. The target dose was prescribed to the 60-70% isodose. (= 6Gy) to allow higher doses within the target volume as for brachytherapy. Subvolumes within the tumour receiving 150-200% of the prescribed dose were allowed (Figures 3 and 4).

#### Treatment planning

The planning CT scan with contrast medium (Ultravist^®^) of the cervix, the pelvic region as well as the lower lumbar spine was performed at least three days after gold marker implantation to allow fiducial consolidation. Two-three days after fiducial implantation, computed tomography (CT) and magnetic resonance imaging (MRI) were performed in the treatment position. Patients emptied rectum and bladder prior to scanning. All patients were placed in the supine position without a vacuum bag or alpha cradle, utilizing a comfortable two-inch foam mat to enhance patient comfort. A knee rest and foot rest were added to stabilize the pelvic region. CT images were acquired with 2-mm slice thickness including the pelvic region as well as the L4 and L5 vertebrae, which is necessary for later DRR generation and spinal alignment. The MRI of the pelvis was conducted (T1 + gadolinium contrast, T2) 2–3 days before starting CyberKnife treatment with the patient in the same position as in the CT scan.

Treatment planning was performed on Multiplan 4.5 (Accuray, Inc.) planning workstations. Inverse treatment planning was used to calculate the optimal dose distribution, applying the maximum dose to the target and restricting the dose to the surrounding structures. Because of the large number of non-isocentric beam directions, the system can deliver complex dose distributions with steep dose gradients (Figure 3). All treatments were planned for five fractions. Target volumes, treatment plans and dose-volume-histograms (DVH) were evaluated by the radiation oncologist (S.M.) and radiologist (B.G.), a specialized CyberKnife expert (M.K.) as well as medical physicists (A.K., R.S.).

#### Treatment delivery

CyberKnife Boost was performed in the 4th-6th week of treatment. Patients were positioned on the robotic treatment couch in the same position as during the planning CT and MRI, with knee- and foot-rests on the 2-inch foam mat. To optimize the initial treatment alignment a so-called “Xsight spine setup plan” was generated, defining the lower part of the lumbar spine as the tracking target. With this setup plan the patient can be positioned according to bony structures of the spine. By moving the robotic treatment couch the patient is aligned to correct the translational as well as rotational positioning deviations. This step is important because of the difficulties in proper fiducial placement in and around the cervical target. If the fiducials are placed too close to each other or the angle between fiducials is too small, a three dimensional tracking structure cannot be defined by the three fiducials. In this situation the correction of rotational positioning deviations is not possible. Therefore the initial setup with spinal tracking offers the possibility to correct rotational errors upfront by couch movements. After the spinal alignment the actual treatment plan is loaded, and the patient is moved to the true treatment position. Using translational table movements, which are previously calculated from the relocation of the treatment center in the planning software, the patient is moved to the treatment position avoiding any changes in rotation. The inter-fraction interval was at least 72 hours.

#### Clinical follow-up

Acute treatment-related toxicity was documented once weekly according to CTCAEv.4.03 [13]. Three months after treatment all but one patient underwent intra-cervical curettage to exclude residual tumor. One patient refused and was followed up clinically.

#### Data analysis

The doses to the PTV, subvolumes of 1 cc and 2 cc (D1cc and D2cc) of the bladder wall, rectal wall and sigmoidal wall were calculated as 2-Gy equivalent doses (EQD2) using the linear quadratic model with α/β = 3 Gy for bladder and sigmoidal wall, α/β = 5 Gy for the rectal wall and α/β = 10 Gy for the cervical tumour, respectively. The EQD2 values of the radiosurgical CyberKnife boost were added to median EQD2 doses of the external beam radiation [2]. For DVH analysis the parameters in Table 1 were calculated. Parameters assessing the PTV coverage and conformity and doses to bladder, rectal, and sigmoidal wall were computed to determine how well we were able to meet dose requirements and constraints with the CyberKnife relative to brachytherapy. To describe the quality of dose distribution to the target CN and CI were used. To demonstrate the quality of the target coverage in relation to the surrounding organs at risk and its dose gradient to the sigmoid and rectum, COIN rectal wall and COIN sigmoidal wall were calculated [14]–[16].

**Table 1:** Structures, DVH parameters, and equations used in parameter calculations

**Structure**	**Parameters**
PTV	Volume of the PTV (VPTV, cc), mean dose (Dmean, Gy), minimal dose covering the PTV (Dmin), the coverage (%), Conformity Index (CI), Conformity Number (CN) [11,12].
Bladder wall	Volume (Vol., cc), D1cc and D2 cc (Gy)
Rectal/sigmoidal wall	Vol. (cc), mean dose (Gy), D1cc, D2cc as recommended by the GEC ESTRO Group [1,2], Conformal Index (COIN) and the volume covered by the prescribed dose (Vref, Gy) [13]
Small bowel	Mean dose from CyberKnife (Dmean, Gy)
**Calculations**[11]–[13]	
Conformal index (COIN)	COIN = CN * ∏ (1 - VORref, i/VOR, i)
	CN) = 1/nCI;
Conformity index (nCI)	nCI = (Vref * PTV)/PTVref^2

## Results

The whole treatment was completed within 45–57 days (median 50 days) in all patients including EBRT, simultaneous chemotherapy and CyberKnife treatment. There was no prolongation of treatment and no therapy break. The EQD2 of EBRT and the CyberKnife boost summed to at least 90 Gy for the tumour. The CyberKnife boost was performed 1–2 times per week with an inter-fraction interval of at least 72 hours. Median PTV volume was 48.9 cc (31.5-68.8 cc). With the prescribed 60-70% covering isodose, Dmean_PTV_ ranged from 33.6-40 Gy, median 36.7 Gy with a coverage of the PTV calculated to 100% of the prescribed dose ranging from 93.0-99.3% (median 97.7%). For the PTV the median CN was 0.78 (range, 0.66 to 0.87) and the median CI was 1.28 (range, 1.15 to 1.52). DVH data for the PTV are summarized in Table 2. Volume of the organs at risk, mean doses, D1cc (EQD2), D2cc (EQD2) and conformity indices are shown in Table 3. The CyberKnife boost added a mean dose of 1.3-7.6 Gy (median 3.6 Gy) to the small bowel over the EBRT mean dose to the small bowel (< 29 Gy). The volume covered by the reference dose was 0.0 cc in ten patients and 0.1 cc for the rectal wall and 0.0 cc, 0.1 cc and 0.3 cc for nine, one and one patient for the sigmoidal wall, respectively. For EBRT D2cc(EQD2) for the bladder wall, rectal wall and sigmoidal wall ranged from 39.47 to 57.12 Gy (median 55.10 Gy); 38.86-54.21 Gy (median 49.83 Gy); and 37.06-51.36 Gy (median 48.67 Gy), respectively. All D1 cc and D2cc(EQD2) values for CyberKnife boost are shown in Table 2. D2cc(EQD2) (CyberKnife) were added to the D2cc(EQD2) levels from EBRT reported above. Intra-cervical curettage confirmed a histological complete response in 9/11 patients. One patient refused curettage and underwent gynecologic examinations and PAP-smears and MRI, which showed no residual tumor. In one patient there was a suspicious residual tumor in the curettage. Uneventful secondary extrafascial hysterectomy was performed which could not confirm residual tumor in the cervix after pathological examination of the specimens.

**Table 2:** DVH Parameters

	**DVH Parameters PTV**								
Pts.	**Planning System Version**	**Prescribed Isodose (%)**	**Total MU (5 Fractions)**	**Dmax (Gy)**	**Dmean (Gy)**	**PTV Coverage (%)**	**V**_**PTV **_**(cc)**	**nCI**	**CN**
1	MultiPlan 4.0.1 [4044]	70	46703	4225.35	36.7	97.7	66.8	1.25	0.80
2	MultiPlan 4.0.2 [4048]	70	48903	4285.71	36.6	94.6	68.8	1.35	0.74
3	MultiPlan 4.0.2 [4048]	70	45268	4285.71	36.6	98.8	43.8	1.37	0.73
4	MultiPlan 4.0.2 [4048]	70	34333	4285.71	34.2	95.6	43.2	1.24	0.81
5	MultiPlan 4.0.2 [4048]	70	47656	4285.71	33.6	95.2	31.5	1.28	0.78
6	MultiPlan 4.0.2 [4048]	70	44361	4285.71	36.3	99.0	43.9	1.20	0.83
7	MultiPlan 4.5.0 [4547]	60	36255	4285.41	38.4	99.3	39.3	1.15	0.87
8	MultiPlan 4.5.0 [4547]	60	36867	5000.00	37.5	97.7	53.5	1.17	0.85
9	MultiPlan 4.5.0 [4547]	60	19845	5000.00	37.6	96.0	48.9	1.28	0.78
10	MultiPlan 4.5.0 [4547]	60	42590	5000.00	40.0	98.4	60.7	1.36	0.74
11	MultiPlan 4.5.0 [4547]	60	50545	5000.00	38.4	93.0	51.4	1.52	0.66

Planning System Version used, prescribed isodose (covering isodose = 6 Gy); maximum dose (Dmax, Gy); mean dose (Dmean, Gy); PTV covered by x% of the dose (PTV coverage, %); PTV volume (cc), CN (Conformity Number); nCI.

**Table 3:** Dose volume parameters for the PTV and the organs of risk

**Pt.**	**PTV**		**Bladder wall**				**Rectal wall**						**Sigmoidal wall**					
No.	Vol. (cc)	D_min _(Gy)	Vol. (cc)	Mean Dose (Gy)	EQD2 (Gy) D1cc	EQD2 (Gy) D2cc	Mean Dose (Gy)	Vol. (cc)	EQD2 (Gy) D1cc	EQD2 (Gy) D2cc	V_ref _(cc)	COIN	Mean Dose (Gy)	Vol. (cc)	EQD2 (Gy) D1cc	EQD2 (Gy) D2cc	V_ref _(cc)	COIN
1	66.8	24.8	18.1	12.0	35.94	32.09	6.20	26,00	20.18	17.78	0,0	0,80	1.10	8.50	1.00	1.0	0.0	0.80
2	68.8	20.8	45.3	10.5	39.48	37.19	7.10	32,30	21.08	19.40	0,0	0,74	12.80	10.20	30.01	22.20	0.0	0.74
3	43.8	19.0	10.8	6.40	8.38	7.61	5.10	10,90	10.72	7.66	0,0	0,73	6.30	16.30	23.35	14.20	0.0	0.73
4	43.2	26.1	9.0	15.9	45.46	34.96	7.50	11,90	38.77	29.35	0,0	0,81	7.40	12.80	29.33	16.10	0.0	0.81
5	31.5	26.6	19.5	9.10	25.00	18.73	7.20	7,70	24.07	7.08	0,0	0,78	9.80	10.80	34.72	20.20	0.0	0.78
6	43.9	26.7	12.6	13.0	40.52	31.62	6.00	28,10	18.26	11.05	0,0	0,83	9.20	5.20	18.00	9.00	0.0	0.83
7	39.3	28.0	17.5	10.6	48.44	36.19	9.50	5,20	21.94	8.26	0,1	0,87	9.10	16.10	28.89	17.10	0.3	0.86
8	53.5	23.6	21.8	10.3	42.37	31.39	5.20	13,70	22.99	12.27	0,0	0,85	3.70	16.70	6.75	5.40	0.0	0.85
9	48.9	19.1	14.9	12.2	41.31	27.78	7.10	23,80	24.07	20.91	0,0	0,78	11.40	18.00	41.57	32.8	0.1	0.78
10	60.7	23.2	21.7	11.3	44.54	35.94	6.50	11,90	19.90	15.00	0,0	0,74	8.60	11.00	19.09	16.10	0.0	0.74
11	51.4	13.7	47.3	9.30	55.81	46.19	8.10	10,10	22.29	14.70	0,0	0,66	6.30	20.60	18.91	15.70	0.0	0.66

*Pt*. = patient, *No*. = number; *PTV *= planning target volume; *Vol*. = Volume (cc); *D*_*min *_= minimum dose; *EQD2 *= biologically equivalent dose for 2Gy fractionation; *D*_*1cc *_= Dose (Gy) to 1 cc oft the organ wall(bladder, rectum, sigmoid); *D*_*2cc *_= Dose (Gy) to 2 cc of the organ wall; *V*_*ref*_. = volume (cc, of a organ at risk) covered by the reference dose (30 Gy); *COIN *= Conformal Index.

Acute toxicity was documented weekly in all patients according to CTCAEv.4.03 [14] and is shown in Table 4.

**Table 4:** Acute treatment-related toxicity (CTCAEv4.03c)

**Acute toxicity**	**Grade 0**	**Grade 1**	**Grade 2**	**Grade 3**	**Grade 4**
Haematologic					
Anaemia	1	1	7	2	0
Thrombocytopenia	1	2	6	3	0
Leukocytopenia	0	4	5	0	1
Gastrointestinal	0	9	2	0	0
Genitourinary	0	9	2	0	0
Vagina	0	11	0	0	0

## Discussion

Brachytherapy plays an important role in the treatment of cervical cancer. However, its cylindrical dose distribution leads to either considerable underdosing in laterally extended, asymmetric tumours or overdosing in order to insure acceptable coverage of the tumor. With implementation of MRI-based brachytherapy boost it might be possible to improve local control rates while decreasing therapy-related toxicity [1,2]. Nevertheless, the MRI-based brachytherapy technique is technically challenging, invasive and time-consuming. Patterns of care analysis for U.S. found only 1% utilization in community practices and only 25% of all responders [3].

Highly sophisticated external beam techniques have been evaluated in gynaecologic tumours [5]–[10]. Mollá et al. [6] found that the use of IMRT to deliver a final boost to areas at high risk for relapse was feasible, well tolerated, and may be considered an acceptable alternative to brachytherapy. The data is not comparable to others, because 14/16 patients had prior surgery and thus the treatment was indicated as part of adjuvant therapy to reduce the risk of recurrence. Hsieh et al. [5] published a case report on helical tomotherapy instead of brachytherapy for the primary treatment for cervical cancer patients with six fractions of 4 Gy up to a total dose of 24 Gy. All patients underwent secondary hysterectomy. However, after a 14-month follow-up one patient presented with lower GI bleeding. Oncological results were encouraging, however, with 96% and 95% overall survival and loco-regional control.

Although different concepts are not comparable, some authors compared stereotactic radiotherapy (SRT) and brachytherapy for cervical cancer patients and found that SRT yielded a significantly better target coverage; dose distributions for critical organs were similar in both types of plans. Some significant differences were also found in maximum doses received by a 2 cc volume of the bladder in favour of SBRT plans [7]–[10]. In the present study we used the same institutional dose concepts for CyberKnife^®^ planning as we do for brachytherapy.

Treatment-related toxicity of innovative concepts should be assessed critically. For traditional dose concepts and the combination of EBRT with brachytherapy, the incidence of major late sequelae of RT in patients with cervical cancer of FIGO stage IIB and III ranges from 10% to 15% [2]. Doses of 75 to 80 Gy delivered in limited volumes by a combination of external beam irradiation and intracavitary therapy result in an incidence of grade 2 and 3 complications below 5% [17,18]. With the application of higher doses, the incidence of complications increases. Applying a conventionally fractionated EBRT boost, late grade 3 toxicity has been observed in 2% of patients and late grade 1 and 2 bowel and bladder toxicities in 41% of patients [4].

Because of the beam directions for robotic radiosurgery, the dose contribution to the small bowel should be observed since late GI toxicity impacts quality of life. In our EBRT series for primary chemo-radiation in cervical cancer patients, we defined a mean dose of ≤ 29 Gy and a V45Gy < 15% as main EBRT dose constraints for the small bowel. In combination with brachytherapy we reported on Grade 3 GI toxicity in 5% of the patients. With CyberKnife boost, the median dose to the small bowel was 3.6 Gy, which was in the range of brachytherapy treatment in our series.

There has been no systematic evaluation of toxicity for patients with cervical cancer who were treated with an IMRT or SRT boost instead of brachytherapy. Few data are available with regard to SRT. Hsieh et al. reported grade 1 nausea and vomiting during treatment [5]. Mollá et al. reported grade 3 rectal bleeding in a previously irradiated patient at a median follow-up of 12.6 months [6]. In a follow-up publication, Jorcano et al. [10] reported on a high rate of acute toxicities (23% GU and 25% GI) of RTOG grade 3 or less.

For MRI-guided brachytherapy, a recent report underlines the predictive value of certain doses to small sub-volumes. Georg et al. showed a 5% and 10% risk for grade 2–4 late effects to the rectum, sigmoid and bladder when increasing the EQD2 in 2 cc of the organ wall from 67–78 Gy for the rectum and 70–101 Gy for the bladder, respectively, whereas no reliable dose relationship could be established for the sigmoid [2].

Considering the EQD2 for EBRT and CyberKnife treatment together, we demonstrated a median EQD2 for the bladder wall in 1 cc and 2 cc of 98.81 Gy and 87.1 Gy, which is correlated with a risk < 10% for grade 2–4 late toxicity according to the estimation from brachytherapy [3]. Similar results were shown for the rectal wall. The expected grade 2–4 GI late toxicity is about 5% at a median EQD2 for 1 cc of the rectal wall of 72.34 Gy (50.4 Gy EBRT plus 21.9 Gy boost) and for 2 cc of the rectal wall 64 Gy (49.8 Gy EBRT plus 14.7 Gy boost). For the sigmoidal wall there is no widely accepted dose-volume predictor of the incidence or severity of GI late toxicity [3]. The low incidence of acute toxicity in the present study is encouraging. The steep dose gradient is expressed by the COIN, which was computed to get an impression of the treatment quality of the CyberKnife independently of homogeneity. Because of the close relationship between the rectum and PTV as well as the sigmoid and PTV we analysed both CN and COIN. Similar values for COIN and CN reflect that the CyberKnife is able to realize very steep dose gradients towards the rectal and sigmoid wall, without compromising the minimal dose to the target volume or coverage [14]–[16].

A comparison of the only available publication on CyberKnife boost for cervical cancer patients [11] is complicated by the fact that, in that study, patients received a range of EBRT and CyberKnife boost doses. Dose constraints and DVH parameters were not well defined, and no calculation of the EQD2 was shown. The dose constraints for the bladder and rectum (V75 Gy) are not accepted parameters which correlate with reported toxicity [11]. Sigmoid doses were not reported. The response was evaluated only clinically. Mild GI and GU toxicity was comparable to our data.

Because of the innovative concept used here, we performed cervical curettages in 10/11 patients (one patient refused) 3 months after treatment in order to verify freedom from local tumour recurrence. No other study provided histological confirmation of freedom from local tumour. Nine of our patients were free of local tumour recurrence after first curettage. In one patient the pathologist described vital tumour in two further consecutive curettages. Therefore the patient underwent a secondary hysterectomy. Surprisingly, in the cervix specimen there was no residual tumour. One patient who refused curettage is tumour free as evaluated by clinical examination as well as PAP smear and MRI. No patient recurred locally at a median follow up of 6 months. GI and GU toxicity was in the range of the standard treatment in our institution. Evaluation of late toxicity requires a longer follow-up.

On the basis of these preliminary results, robotic radiosurgery emulating the standard brachytherapy boost in patients with cervical cancer seems to be feasible and did not lead to a higher incidence of acute toxicity compared to the institutional standard of IMRT/RapidArc in combination with MRI-based brachytherapy. Internationally accepted dose constraints for MRI-based brachytherapy could be fulfilled applying the CyberKnife technique in all patients with locally advanced tumours and a wide range of PTV volumes.

## Competing interest

The authors declare that they have no competing interest.

## Authors’ contributions

SM: Idea, concept, patients´ acquisition, contouring, plan evaluation, manuscript writing, revision, submission. CK: Patients´ acquisition, laparoscopic staging, follow up examination, manuscript discussion. VB: Manuscript reading. AK: Planning procedure. ON: Planning procedure, manuscript discussion. WW: Discussion and revising the manuscript. BG: Target volume delineation, MRI. MK: Planning, treatment delivery, discussion, writing (treatment part). All authors read and approved the final manuscript
